# Choosing important health outcomes for comparative effectiveness research: an updated review and user survey

**DOI:** 10.1186/1745-6215-16-S3-P8

**Published:** 2015-11-24

**Authors:** Sarah L Gorst, Elizabeth Gargon, Paula R Williamson

**Affiliations:** 1 Department of Biostatistics, University of Liverpool, Liverpool, UK

## Background

A COS represents an agreed minimum set of outcomes that should be measured and reported in all trials of a specific condition. The COMET (Core Outcome Measures in Effectiveness Trials) initiative aims to collate and stimulate the development and application of COS, by including data on relevant studies within a publically available internet-based resource. In recent years, there has been an interest in increasing the development of COS. Therefore, this study aimed to provide an update of a previous review, and examine the quality of development of COS. A further aim was to understand the reasons why individuals are searching the COMET database.

## Methods

A multi-faceted search strategy was followed, in order to identify studies that sought to determine which outcomes/domains to measure in clinical trials of a specific condition. Additionally, a pop up survey was added to the COMET website, to ascertain why people were searching the COMET database.

## Results

Thirty-two reports relating to 29 studies were eligible for inclusion in the review. There has been an improvement in the description of the scope of a COS and an increase in the proportion of studies using literature/systematic reviews and the Delphi technique. Clinical experts continue to be the most common group involved in developing COS, however patient and public involvement has increased. The pop-up survey revealed the most common reasons for visiting the COMET website to be thinking about developing a COS and planning a clinical trial.

## Conclusions

This update demonstrates that recent studies appear to have adopted a more structured approach towards COS development and public representation has increased. However, there remains a need for developers to adequately describe details about the scope of COS, and for greater public engagement. The COMET database appears to be a useful resource for both COS developers and users of COS.

